# Nationwide Genomic Data Analysis of Japanese Prostate Cancer Patients From C‐CAT Database

**DOI:** 10.1002/cam4.71085

**Published:** 2025-07-26

**Authors:** Shigehiro Tsukahara, Masaki Shiota, Shohei Nagakawa, Tokiyoshi Tanegashima, Satoshi Kobayashi, Takashi Matsumoto, Masatoshi Eto

**Affiliations:** ^1^ Department of Urology, Graduate School of Medical Sciences Kyushu University Fukuoka Japan

**Keywords:** cancer, C‐CAT, CGP, comprehensive genome profiling, prostate

## Abstract

**Purpose:**

Prostate cancer is a leading malignancy among men, and genomic alterations are known to impact disease progression and treatment response. However, racial and ethnic differences may influence genomic profiles, necessitating population‐specific analyses. This study aimed to characterize the genomic landscape and its clinical significance in Japanese patients with treatment‐resistant, unresectable prostate cancer using data from the Center for Cancer Genomics and Advanced Therapeutics (C‐CAT) database.

**Methods:**

We analyzed data from patients with advanced or metastatic prostate cancer who had progressed after standard therapies and underwent comprehensive genomic profiling between 2019 and 2022. We assessed the frequency of genomic alterations, tumor mutation burden (TMB), and microsatellite instability (MSI) status. Associations between genomic features and clinical outcomes were also examined.

**Results:**

A total of 2634 patients were included. Family history was reported in 12.5% for prostate cancer, 1.5% for breast cancer, 5.2% for pancreatic cancer, and 1.1% for ovarian cancer. *AR* gene alterations were observed in 18% of patients. *TP53* and *BRCA2* mutations were identified in 34% and 12% of cases, respectively. Mutations in *TP53*, as well as alterations in genes related to the cell cycle, epigenetic regulation, MYC signaling, and the PI3K pathway, were associated with poorer overall survival.

**Conclusions:**

This study provides a comprehensive overview of genomic alterations in advanced prostate cancer among Japanese patients and identifies key mutations linked to prognosis. These findings highlight the value of personalized prognostic assessment based on genomic profiling to guide clinical decision‐making in this population.

## Graphical abstract

This study analyzed genomic data from 2634 Japanese patients with advanced prostate cancer using the C‐CAT database, revealing frequent alterations in genes such as *TP53*, *BRCA2*, and *AR*. Key mutations, particularly in TP53 and pathways like PI3K and MYC signaling, were linked to poorer survival, underscoring the importance of population‐specific genomic profiling for personalized treatment strategies.

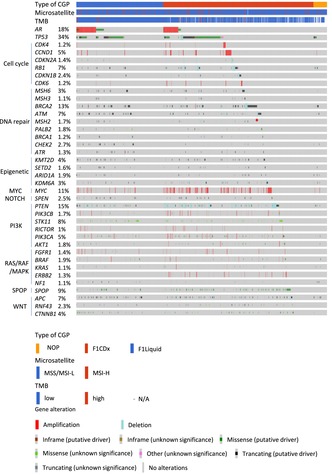

**Abbreviations**
*AR*
: androgen receptor
C‐CAT: Center for Cancer Genomics and Advanced Therapeutics
MSI: microsatellite instability
TMB: tumor mutation burden

## 1 Introduction

Prostate cancer was the second most commonly diagnosed malignancy among men worldwide in 2020, with an estimated 1.4 million new cases and approximately 375,000 deaths [1]. It is estimated that one in six men will be diagnosed with prostate cancer during their lifetime [1]. Although prostate cancer is generally associated with relatively long‐term survival and often considered a disease at risk of overtreatment [2], it remains the second leading cause of cancer‐related deaths in men globally. In Japan, the incidence of prostate cancer is also notably high. According to a 2023 national survey, prostate cancer accounted for 98,600 new cases, comprising 16.7% of all cancers and ranking as the most frequently diagnosed cancer in Japanese men. Despite its typically indolent nature, prostate cancer was the sixth leading cause of cancer‐related mortality among Japanese men, with approximately 14,000 deaths reported in a single year [3].

Mutations in DNA repair genes, including *BRCA1* and *BRCA2*, are associated with sensitivity to poly(ADP‐ribose) polymerase (PARP) inhibitors, and are now recognized as valid therapeutic biomarkers in several clinical guidelines. However, notable differences in prostate cancer incidence and clinical outcomes have been reported among racial and ethnic groups [4, 5], emphasizing the importance of analyzing genomic data specific to the Japanese population.

Following pivotal findings from The Cancer Genome Atlas (TCGA) and other large‐scale genomic studies, cancer genome research in Japan has rapidly advanced. Since 2018, the Ministry of Health, Labour, and Welfare has designated 12 core hospitals and 461 affiliated hospitals across the country as centers for cancer genomic medicine. In 2019, comprehensive genomic profiling (CGP) was approved for national insurance coverage in Japan and is now routinely performed for patients with advanced malignancies who have completed standard treatments, including chemotherapy. Genomic diagnostics using CGP play a critical role in guiding therapeutic decision‐making in clinical practice.

The Center for Cancer Genomics and Advanced Therapeutics (C‐CAT) database was established by the Ministry of Health, Labour and Welfare in Japan. C‐CAT collects the results of CGP assays conducted under the national health insurance system, along with clinical information from patients diagnosed with advanced malignancies, including cancers of unknown primary origin and rare cancers. Based on its proprietary Cancer Knowledge Database, as well as commercial resources such as AQAIGEN Clinical Insight and the JAX Clinical Knowledgebase (JAX‐CKB), C‐CAT provides clinical annotations of CGP results. These CGP results and annotations are reviewed by expert panels at designated core and affiliated hospitals. The panels evaluate the findings and provide feedback to the primary physicians, including information on potential therapeutic agents and relevant clinical trials for which the patient may be eligible [6]. In addition to its clinical utility, the C‐CAT database also serves as a valuable resource for research by offering access to a large‐scale dataset of malignancies. The database includes not only genomic data but also a wide range of clinical and demographic patient information. Upon approval of a formal application, researchers can utilize this database to study advanced and rare cancers in the Japanese population.

In this study, we aimed to analyze and report the genomic landscape and its prognostic relevance in Japanese patients with prostate cancer using data from the C‐CAT database.

## 2 Materials and Methods

### 2.1 Database of the C‐CAT

This study was conducted using the C‐CAT database established by the Ministry of Health, Labour, and Welfare in Japan. The C‐CAT collects the results of CGP assays under the health insurance system and clinical information of patients [7]. CGP assays were conducted using three types of diagnostic tools in Japan: FoundationOne CDx (F1CDx) (Foundation Medicine Inc., Cambridge, MA, USA), FoundationOne Liquid CDx (F1Liquid) (Foundation Medicine Inc.) and OncoGuide NCC Oncopanel System (NOP) (Sysmex Co. Ltd., Hyougo, Japan). All these assays cover over 100 cancer‐related genes.

These assays are used for patients with various malignancies who have completed or are expected to complete standard chemotherapy. By October 2024, the number of patients who underwent CGP assays reached 89,469, all of whom were registered in the C‐CAT database. Among them, 99.6% consented to the secondary use of their registered data [8].

The C‐CAT database includes patient information (e.g., age, sex), disease information (e.g., cancer type, histology, metastatic sites), sample conditions (e.g., date, method, and site of collection), treatment information (e.g., chemotherapy regimens, adverse events), and clinical outcomes.

### 2.2 Patients

We retrospectively analyzed patients with advanced or metastatic prostate cancer who performed CGP assays between July 2019 and December 2022 registered in the C‐CAT database. We collected the following data as a patient's background: patient information (age, smoke history, family history of malignant tumor associated with *BRCA* mutation, Eastern Cooperative Oncology Group performance status; ECOG‐PS), malignancy information (Gleason grade group, metastasis lesion, outcomes), treatment information (the number of treatment received before CGP, treatment drug, the response for treatment and overall response rate; ORR), assay information (type of CGP, samples of CGP).

### 2.3 Gene Alteration

As the gene alteration, we analyzed single nucleotide variants (SNVs), insertion and deletion (INDEL), copy number alteration (CNA), heterozygous loss (HETLOSS), tumor mutation burden (TMB), and microsatellite instability (MSI). We analyzed genomic alteration defined as “Oncogenic” or “Likely Oncogenic”.

Oncogenic alterations were grouped by major signaling pathways from pan‐cancer analyses, as below. Gene groups related to the cell cycle were defined by alterations in 10 genes (*CCND1, CCNE1, CDK4, CDK6, CDKN1A, CDKN1B, CDKN2A, CDKN2B, CDKN2C* and *RB1*). DNA repair defects were defined by alterations in 24 genes, including Homologous Recombination DNA Repair (HRR) and Mismatch Repair (MMR) genes (*ATM, ATR, BIRD, BRCA1, BRCA2, BRIP1, CHEK1, CHEK2, ERCC4, FANCA. FANCC, MLH1, MRE11, MSH2, MSH3, MSH6, NBN, PALB2, PARP1, PMS2, POLE, RAD51, RAD52* and *XRCC2*). Epigenetic‐related gene alterations were defined by alterations in 5 genes (*ARID1A, KDM6A, KMT2A, KMT2D* and *STED2*). The Notch pathway genes were defined by 8 genes (*CREBBP, EP300, FBXW7, KDM5A, SPEN, NOTCH1, NOTCH2* and *NOTCH3*). PI3K pathway genes were defined by 12 genes (*AKT1, AKT2, AKT3, INPP4B, MTOR, PIK3CA, PIK3CB, PTEN, RICTOR, STK11, TSC1* and *TSC2*). The RAS/RAF/MAPK pathway included 25 genes (*ABL1, ALK, BRAF, CBL, EGFR, ERBB2, ERBB3, ERBB4, FGFR1, FGFR2, FGFR3, FGFR4, FLT3, HRAS, IGF1R, IRS2, JAK2, KIT, KRAS, MAP2K1, MAPK1, MET, NF1, NRAS* and *NTRK1*). The WNT signaling pathway consisted of 6 genes (*AMER1, APC, AXIN1, CTNNB1, GSK3B* and *RNF43*). *TP53* was not grouped with DNA repair genes and was considered separately, as were two individual genes relevant to prostate cancer (*AR, SPOP*) [9].

Written informed consent was obtained from all patients. The study was conducted in accordance with the principles described in the Declaration of Helsinki and the Ethical Guidelines for Epidemiological Research enacted by the Japanese Government. The study protocol was approved by Kyushu University Hospital Institutional Review Board (approval number 22028‐00) and the C‐CAT review board (CDU2022‐033E02).

### 2.4 Statistical Analyses

All statistical analyses were performed using EZR software (Saitama Medical Center, Jichi Medical University, Saitama, Japan), a graphical user interface for R 2.13.0. Statistical analyses related to categorical variables were performed using Fisher's exact test. For analysis of overall survival (OS), death from any cause was defined as the end event. Patients with none of these events were censored at the last follow‐up visit. All *P*‐values are two‐sided. *p* < 0.05 were considered statistically significant.

## 3 Result

### 3.1 Patient Characteristics

A total of 2634 patients with advanced or metastatic prostate cancer were included in this study (Table 1). The median age at the time of CGP testing was 72 years (range, 23–93 years). Gleason grade group information was available for only 144 patients (5.5%), among whom 63.89% were diagnosed with Gleason grade group 5. Regarding family history of cancers associated with *BRCA* mutations, 329 patients (12.5%) reported a family history of prostate cancer, 40 (1.5%) of breast cancer, 138 (5.2%) of pancreatic cancer, and 29 (1.1%) of ovarian cancer.

**TABLE 1:** Patients characteristics.

		*n* = 2634
Age (range)		72 (23–93)
Gleason grade group (%)	1	0 (0.0)
	2	6 (0.2)
	3	17 (0.6)
	4	29 (1.1)
	5	92 (3.5)
	NA	2490 (94.5)
Smoke history (%)	+	1548 (58.8)
	−	799 (30.3)
	NA	287 (10.9)
Family history of prostate cancer (%)	+	329 (12.5)
	−	2305 (87.5)
Family history of breast cancer (%)	+	40 (1.5)
	−	2594 (98.5)
Family history of pancreas cancer (%)	+	138 (5.2)
	−	2496 (94.8)
Family history of ovarian cancer (%)	+	29 (1.1)
	−	2605 (98.9)
ECOG PS (%)	0	1617 (61.4)
	1	800 (30.4)
	2	92 (3.5)
	3	19 (0.7)
	4	1 (0.0)
	NA	105 (4.0)
Metastasis at registeration (%)	+	2462 (93.5)
	−	97 (3.7)
	NA	75 (2.8)

Abbreviations: ECOG PS, East Clinical Oncology Group performance status; NA, not available.

### 3.2 Type and Sample of CGP Test

In this study, the most commonly used comprehensive genomic profiling (CGP) assay was F1CDx, which was performed in 1576 patients (59.8%), followed by F1Liquid in 911 patients (34.6%) and NOP in 146 patients (5.5%) (Table 2). Among the 2634 patients, 1425 (54.1%) were evaluated using primary tumor tissue, while 295 patients underwent biopsy of metastatic lesions. Details of the metastatic sites are listed in Table 2. At the time of CGP testing, 93% of patients had confirmed metastatic disease, with the majority diagnosed with metastatic castration‐resistant prostate cancer (mCRPC). Only 3.7% of patients had no detectable metastatic lesions and were classified as having nonmetastatic castration‐resistant prostate cancer. Most patients had received multiple lines of hormonal therapy and chemotherapy prior to CGP testing (Table 2).

**TABLE 2:** Information of CGP test and the details of the treatment before and after CGP test.

		*n* = 2634
CGP test (%)	FoundationOne CDx	1576 (59.8)
	FoundationOne Liquid	911 (34.6)
	NCC OncoPanel	146 (5.5)
	NA	1 (0.0)
Sample for CGP test (%)	Primary	1404 (53.3)
	Biopsy	1122 (42.6)
	Prostatectomy	281 (10.7)
	Transurethral surgery	1 (0.0)
	Metastatic lesion	316 (12.0)
	Bladder	86 (3.3)
	Lymph node	83 (3.2)
	Bone	45 (1.7)
	Liver	35 (1.3)
	Lung	26 (1.0)
	Soft tissue	8 (0.3)
	Adrenal grand	5 (0.2)
	Peritoneum/Pleura	4 (0.2)
	Ureter	4 (0.2)
	Brain	4 (0.2)
	Skin	4 (0.2)
	Muskle	2 (0.1)
	Testis	2 (0.1)
	Intestin	1 (0.1)
	Penis	1 (0.1)
	Other tissue	6 (0.2)
	Blood	911 (34.6)
	NA	3 (0.1)
Number of prior treatment (%)	1	768 (29.2)
	2	1330 (50.5)
	3	112 (4.3)
	4	112 (4.3)
	> 5	174 (6.6)
	NA	138 (5.2)
Prior treatment (%)	Bicalutamide	1186 (45.0)
	Flutamide	398 (15.1)
	Abiraterone acetate	1113 (42.3)
	Enzalutamide	1070 (40.6)
	Apalutamide	198 (7.5)
	Darolutamide	56 (2.1)
	Docetaxel acetate	1279 (48.6)
	Cabazitaxel acetate	736 (27.9)
	Olaparib	10 (0.4)
	Ra223	184 (7.0)
Number of patients recieving treatment after CGP test (%)		230 (8.7)
Treatment (%)	Abiraterone acetate	9 (0.3)
	Enzalutamide	5 (0.2)
	Apalutamide	4 (0.2)
	Darolutamide	2 (0.1)
	Docetaxel acetate	26 (1.0)
	Cabazitaxel acetate	24 (0.9)
	Olaparib	115 (4.4)
	Ra223	5 (0.2)
	Others	40 (1.5)

Abbreviations: CGP, comprehensive genome profiling; NA, not available.

### 3.3 Gene Alteration of Prostate Cancer

**FIGURE 1:**
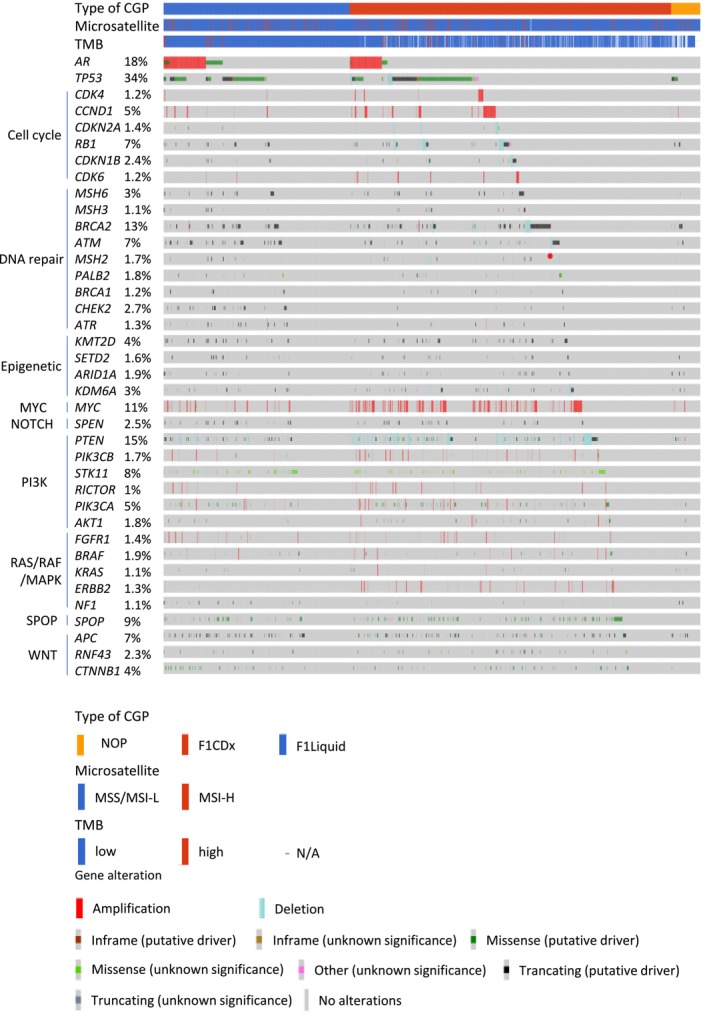
The landscape of comprehensive genome profiling from unresectable treatment‐resistant prostate cancer. The types of gene panel tests performed on 2634 patients, microsatellite status, tumor mutation burden status, and the types of detected genomic alterations were listed according to pathway. CGP, comprehensive genome profiling; F1CDx, FoundationOne CDx; F1Liquid, FoundationOne Liquid CDx; MSI‐H, microsatellite instability‐high; MSI‐L, microsatellite instability‐low; MSS, microsatellite stable; N/A, not available; NOP, NCC Oncopanel System; TMB, tumor mutation burden.

**FIGURE 2:**
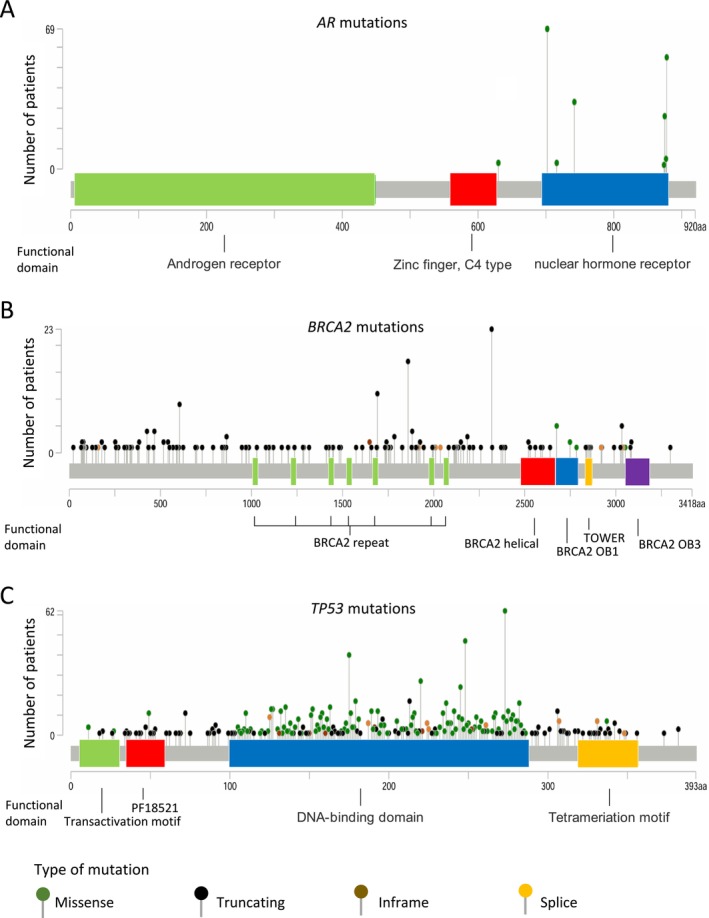
The mapping of mutation spots from frequently mutated genes including *AR* (A), *BRCA2* (B), and *TP53* (C) were shown. The *x*‐axis represents the amino acid position, while the *y*‐axis indicates the number of patients with the same genomic mutation. Colored regions represent domains with critical functional significance, and the color of each dot corresponds to the type of amino acid substitution induced by the mutation.

Clinically significant gene alterations were identified in 2137 of the 2634 patients (81.1%). The major gene alterations and frequently mutated genes are summarized in Figure 1. Alterations in the *AR* gene were observed in 18% of patients, with 377 cases showing *AR* gene amplification (Figure 1). The most frequently detected *AR* point mutations resulting in amino acid substitutions included L702H (70 patients, 2.7%), T878A/S (55 patients, 2.1%), W742C/L (33 patients, 1.3%), and H875Y (26 patients, 1.0%) (Figure 2A). *BRCA2* was also frequently mutated, with alterations detected in 13% of the cohort. Truncating mutations were the predominant alteration type, and recurrent mutations such as R2318* and I1859Kfs*3 were observed in multiple patients (Figure 2B). *TP53* mutations were the most prevalent, detected in 1055 patients. The most frequently altered loci were located at chromosomal positions (GRch38) chr17:7,673,802, chr17:7,674,220, chr17:7,674,221, and chr17:7,675,088 (Figure 2C).

### 3.4 Characteristics of TMB and MSI

**FIGURE 3:**
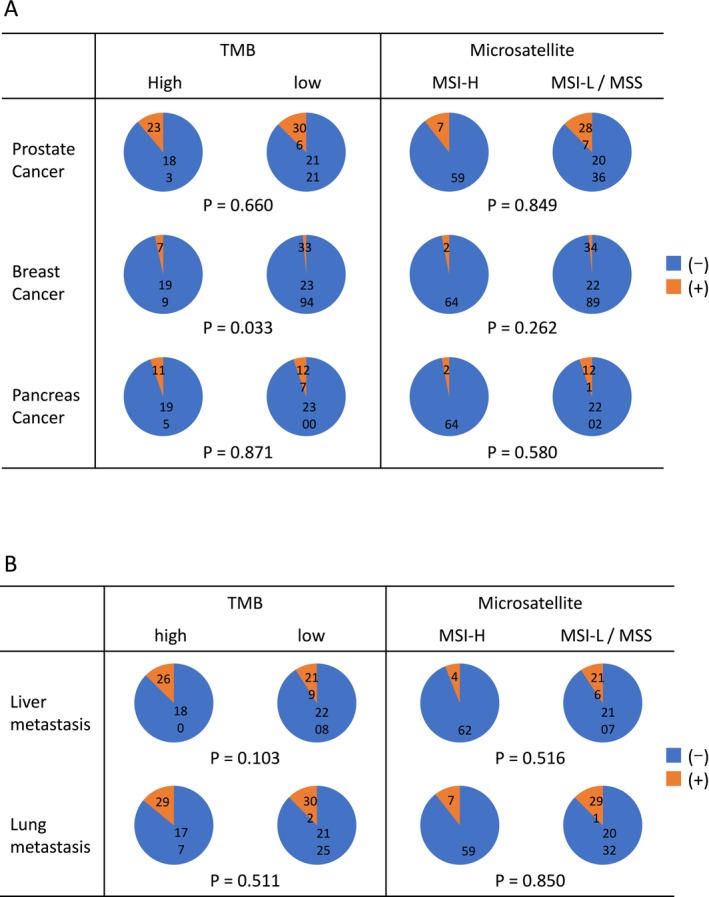
Patients' backgrounds and tumor characteristics. Patients were divided into two groups by tumor mutation burden (TMB) and microsatellite instability. The pie charts show the presence or absence of a family history of malignancy and the presence or absence of organ metastasis, respectively. MSI‐H, microsatellite instability‐high; MSI‐L, microsatellite instability‐low; MSS, microsatellite stable.

Among the 2634 patients included in this study, 206 (7.82%) were classified as having high tumor mutational burden (TMB‐high), while 2427 (92.2%) were categorized as TMB‐low; TMB data were unavailable for one patient. Regarding microsatellite instability (MSI) status, 66 patients (2.51%) were classified as MSI‐high (MSI‐H), and 2323 (88.2%) as MSI‐low or microsatellite stable (MSS). MSI data were not available for 245 patients. The background characteristics of these subgroups are summarized in Figure 3. In terms of family history (Figure 3A), an inverse correlation was observed between TMB‐high status and a family history of breast cancer (*p* = 0.033). In contrast, no significant association was identified between MSI status and any type of familial cancer history.

**FIGURE 4:**
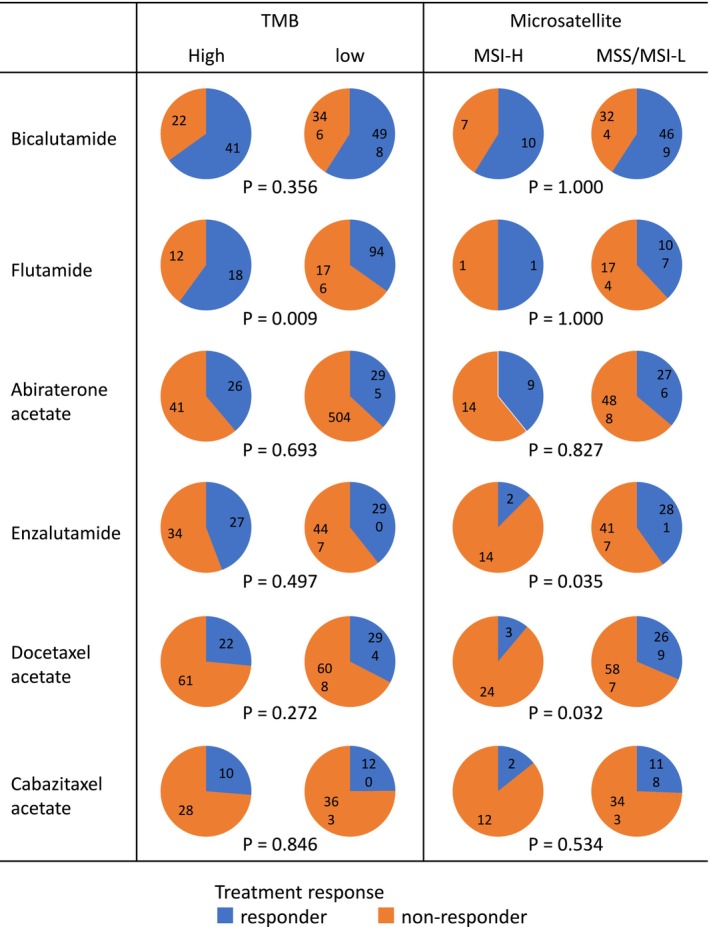
Treatment response and tumor characteristics. Patients were divided into two groups by tumor mutation burden (TMB) and microsatellite instability. The pie charts show the number of responders or non‐responders to each drug. Responders contain complete response (CR) and partial response (PR) for treatment, and non‐responders contain stable disease (SD) and progressive disease (PD), respectively. MSI‐H, microsatellite instability‐high; MSI‐L, microsatellite instability‐low; MSS, microsatellite stable.

We also evaluated the presence of lung and liver metastases as common clinical factors potentially associated with prognosis. However, no significant correlation was found between the presence of these metastases and either TMB or MSI status (Figure 3B). Treatment response was analyzed for each drug by stratifying patients into responder (complete response [CR] + partial response [PR]) and non‐responder (stable disease [SD] + progressive disease [PD]) groups (Figure 4). When stratified by TMB status, a significant difference in response was observed for flutamide (*p* = 0.009), with TMB‐high patients showing greater response rates. For other agents, including apalutamide, darolutamide, olaparib, and radium‐223 (Ra223), the number of treated patients was too small to permit meaningful statistical analysis. Also, due to the limited number of patients in the MSI‐H group, statistical analysis of treatment response by drug based on MSI status was not feasible.

### 3.5 Overall Survivals (OS)

**FIGURE 5:**
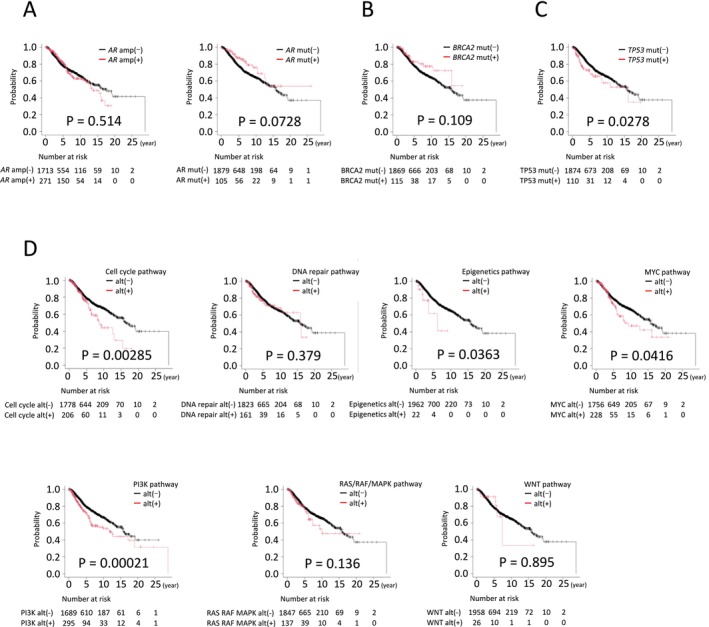
Kaplan–Meier curves for overall survival according to the presence or absence of gene alterations. The overall survival according to *AR* (A), *BRCA2* (B), and *TP53* (C) gene alterations, and signaling pathways indicated (D) were shown. alt, alteration; Amp, amplification; mut, mutation.

The impact of genomic alterations on treatment outcomes was evaluated using Kaplan–Meier survival analyses (Figure 5). For *AR*, a key therapeutic target in prostate cancer, no significant difference in OS was observed between patients with and without *AR* amplification. However, patients harboring *AR* mutations showed a trend toward improved OS compared to those with wild‐type *AR*, although the difference did not reach statistical significance (Figure 5A; *p* = 0.0728). Similarly, *BRCA2* mutations were associated with a non‐significant trend toward longer OS (Figure 5B; *p* = 0.109). In contrast, *TP53* mutations, the most frequent alteration in our cohort, were significantly correlated with shorter OS (Figure 5C).

When genomic alterations were analyzed by pathway, significant differences in OS were observed for alterations in genes involved in cell cycle regulation, epigenetic modulation, MYC signaling, and the PI3K pathway (Figure 5D).

## 4 Discussion

Genomic alterations in prostate cancer influence both treatment strategies and patient prognosis. In this study, we analyzed genomic profiles of Japanese patients with advanced prostate cancer using the C‐CAT database and identified several gene groups associated with clinical outcomes. Mutations in *TP53* were associated with differences in OS. Subgroup analyses further revealed that alterations in genes involved in cell cycle regulation, epigenetic modification, MYC signaling, and the PI3K pathway were significantly correlated with prognosis, suggesting that these genomic pathways may serve as useful prognostic biomarkers.

Recent studies have linked *BRCA* gene mutations to various malignancies, including breast, ovarian, and pancreatic cancers, highlighting the importance of monitoring family histories of these cancers [10, 11, 12, 13]. Furthermore, mutations in DNA damage repair genes such as *BRCA2*, *ATM*, *CHEK2*, *BRCA1*, *RAD51*, and *PALB2* have been implicated in familial prostate cancer [14]. Family history is a well‐established risk factor for prostate cancer, supported by both twin and epidemiological studies [2]. In our C‐CAT‐based analysis, we examined family histories of malignancies, focusing on prostate, breast, pancreatic, and ovarian cancers. Among Japanese patients with metastatic prostate cancer, 12.5% had a family history of prostate cancer, whereas the prevalence of other malignancy types was relatively low (approximately 1%–5%). These findings suggest that, in addition to *BRCA1/2*, other genomic alterations—including polygenic risk variants—may contribute to familial prostate cancer in the Japanese population. However, because F1CDx and F1Liquid do not assess germline alterations, the results should be interpreted with caution in the context of family history. *AR* gene alterations were observed in approximately 18% of cases. It is well established that the frequency of *AR* amplification increases as prostate cancer progresses, with reported rates ranging from 10% to 20% in castration‐resistant prostate cancer (CRPC) and 41%–52% in mCRPC [15, 16, 17, 18, 19, 20]. Among point mutations in the ligand‐binding domain of *AR*, which are commonly associated with CRPC and known to affect treatment efficacy, several mutations were frequently observed in our cohort: L702H (70 patients, 2.7%), T878A/S (55 patients, 2.1%), W742C/L (33 patients, 1.3%), and H875Y (26 patients, 1.0%). Previous studies, including a meta‐analysis of CRPC cases, have reported that L702H (2%–4%), H875Y (2%–5%), and T878A (2%–5%) are the most prevalent mutations [15, 16, 17]. Additionally, W742C and W742L were found at frequencies of 2.3% and 0.5%, respectively [15, 16]. In our study, the observed mutation frequencies were consistent with these prior reports, and we suggest that these mutations may play a role in treatment responsiveness.

*BRCA2* mutations were identified in 12% of patients, a frequency that is consistent with previous reports [18]. Among the *BRCA2* mutations, truncating variants, such as R2318*, were frequently observed, which aligns with findings reported by Momozawa et al. [19]. Notably, the majority of mutations detected in this study were truncating mutations. In addition, a high frequency of *BRCA2* heterozygous loss (HETLOSS) was also observed, both of which are commonly associated with *BRCA2* inactivation. Although *BRCA2* mutations are generally associated with poor prognosis [20], PARP inhibitors such as olaparib and talazoparib have reduced progression risk for patients with *BRCA* mutations. As a result, despite the association with poor outcomes, the availability of targeted therapies may have mitigated the survival disadvantage, leading to no significant difference in OS.

*TP53* was the most frequently observed gene mutation in this study, with mutations detected in 34% of patients. This frequency is consistent with previous reports, which have indicated a rate of 30%–50% [21, 22]. While several recurrent *TP53* mutations were identified, each specific variant occurred in only approximately 1% of cases, suggesting that no single mutation is highly prevalent. This finding was further supported by an analysis of 13,857 prostate cancer cases from 29 studies registered in cBioPortal, where the frequency of each specific *TP53* mutation was similarly below 1% (https://www.cbioportal.org/). *TP53* alterations were associated with worse OS in CRPC patients, which aligns with recent reports describing the aggressive behavior of *TP53*‐altered prostate cancers [21].

Furthermore, a significant association was observed between OS (OS) and several pathways. Alterations in genes related to the cell cycle were strongly associated with prognosis. This finding is consistent with previous reports, including a prognostic scoring system for prostate cancer developed by Silvia et al., based on the expression of cell cycle–related genes [23, 24]. Alterations in epigenetic pathway genes were also associated with poorer outcomes. These genes have been reported to activate downstream gene expression, contributing to prostate carcinogenesis [25, 26]. The PI3K pathway plays a critical role in the proliferation and invasion of prostate cancer [27]. MYC is strongly linked to the progression of prostate cancer through its overexpression and activation [28], and it has been reported that these pathways cooperate with others to further promote malignancy [29]. In this study, patients with alterations in these pathways exhibited significantly worse progression. In cases with multiple genomic alterations, careful consideration should be given to the possibility that individual mutations may influence each other's biological effects.

Since our study focused exclusively on Japanese patients with advanced prostate cancer, we also compared our findings with genomic profiles reported from other regions. A study from the United States, where the population is genetically distinct from the Japanese, reported higher mutation frequencies in *AR* (62.7%), *TP53* (53.3%), and *PTEN* (40.7%) compared to our cohort. In contrast, the frequencies of alterations in cell cycle–related genes, DNA repair genes, and *SPOP* were comparable between Japanese and American patients [30]. In African populations, the mutation frequencies of *AR* (19%), *TP53* (35%), and *BRCA2* (8%) were relatively similar to those observed in our analysis [31]. Meanwhile, a report from Korea, a population considered genetically closer to the Japanese, showed many similar mutation frequencies [32, 33]. However, differences were also observed, including *AR* (42.9%), *KMT2D* (20%), and *SPOP* (25%). These findings suggest that conducting genomic analyses within a given population is important for developing effective, population‐specific treatment strategies.

This study has several limitations. First, there were instances of missing data due to the nature of the database study. Second, treatment decisions were at the discretion of the attending physicians. While this reflects real‐world clinical practice, variations in the sequence of drug administration may have influenced treatment efficacy, requiring careful interpretation of the results. Third, the number of patients in some subgroups was relatively small, limiting the ability to perform robust statistical analyses for these therapies.

This study provides detailed insights into the clinical backgrounds and genomic alterations in Japanese patients with advanced prostate cancer. Genomic profiling and its prognostic implications are of great importance for guiding treatment strategies. Although there are currently limitations in Japan regarding the timing of CGP and access to certain therapies, the expansion of approved indications in the future may enable earlier identification of actionable genomic alterations and the selection of appropriate treatments for patients requiring multidisciplinary care. The accumulation of further cases, especially those with data on treatment response, is expected to advance our understanding and contribute to the improved management of prostate cancer in the Japanese population.

## Author Contributions

**Shigehiro Tsukahara:** formal analysis (equal), investigation (equal), methodology (equal), resources (equal), visualization (equal), writing – original draft (equal). **Masaki Shiota:** conceptualization (equal), methodology (equal), project administration (equal), resources (equal), supervision (equal), writing – original draft (equal). **Shohei Nagakawa:** resources (equal), writing – review and editing (equal). **Tokiyoshi Tanegashima:** resources (equal), writing – review and editing (equal). **Satoshi Kobayashi:** resources (equal), writing – review and editing (equal). **Takashi Matsumoto:** resources (equal), writing – review and editing (equal). **Masatoshi Eto:** supervision (equal), writing – review and editing (equal).

## Ethics Statement

Written informed consent was obtained from all patients. The study was approved by Kyusyu University Hospital Institutional Review Board (approval number 22028‐00). The study was conducted in accordance with the principles described in the Declaration of Helsinki and the Ethical Guidelines for Epidemiological Research enacted by the Japanese Government.

## Conflicts of Interest

Masaki Shiota received honoraria from Janssen Pharmaceuticals, AstraZeneca, Astellas Pharma, Sanofi, and Bayer Yakuhin, as well as research funding support from Astellas Pharma. Masatoshi Eto received honoraria from Ono Pharmaceutical, Takeda Pharmaceuticals, Novartis Pharma, Pfizer, Bristol‐Myers Squibb, Janssen Pharmaceuticals, MSD, Merck Biopharma, AstraZeneca, and Eisai, as well as research funding support from Bayer Yakuhin, Astellas Pharma, Ono Pharmaceutical, and Takeda Pharmaceuticals. The other authors declare no conflicts of interest.

## Data Availability

The data that support the findings of this study are available from C‐CAT. Restrictions apply to the availability of these data, which were used under license for this study.
